# Low-Dose Sublingual Ketamine for the Treatment of Raynaud's Phenomenon

**DOI:** 10.7759/cureus.77449

**Published:** 2025-01-14

**Authors:** Mitchell B Liester

**Affiliations:** 1 Department of Psychiatry, University of Colorado School of Medicine, Colorado Springs, USA

**Keywords:** anti-inflammatory drugs, cold sensitivity, low-dose ketamine, microvascular perfusion, numbness, peripheral vasodilation, raynaud's phenomena, skin color, skin texture, tingling

## Abstract

Raynaud's phenomenon is a vascular disorder characterized by episodic vasospasm of small arteries, primarily affecting the hands and feet. Standard treatment strategies typically include lifestyle modifications to avoid cold exposure and stress, alongside pharmacological interventions aimed at increasing blood flow and reducing vascular constriction. Ketamine, an FDA-approved anesthetic since 1970, exhibits analgesic and vasodilatory properties that may enhance perfusion. This case report describes a woman with primary Raynaud's phenomenon whose symptoms improved significantly during treatment with low-dose sublingual ketamine prescribed for treatment-resistant depression. Further research into the use of this safe and inexpensive medicine as a treatment for Raynaud's phenomenon is recommended.

## Introduction

Raynaud's phenomenon is a vascular disorder characterized by episodic vasospasm of the small arteries, typically in the fingers and toes, that occurs in response to cold or emotional stress. This leads to a triphasic color change in the affected areas: pallor (due to ischemia), cyanosis (from deoxygenated blood), and erythema (as reperfusion occurs) [1]. The condition is more common in colder climates and is also more common in women than men. Non-population-based studies of prevalence show that 3-12.5% of men and 6-20% of women report symptoms of Raynaud's phenomenon [2]. Unlike secondary Raynaud's phenomenon, which is associated with underlying connective tissue diseases such as systemic sclerosis, primary Raynaud's phenomenon occurs without a known underlying pathology and is considered benign [3]. Management focuses on avoiding triggers, such as cold exposure and stress, and pharmacological interventions that are intended to improve blood flow and reduce vascular constriction, such as calcium channel blockers [2,4].

Ketamine is a dissociative anesthetic and N-methyl-D-aspartate (NMDA) receptor antagonist that has gained prominence for its diverse therapeutic applications. Initially developed for anesthesia, this medicine induces sedation and pain relief by blocking excitatory glutamate signaling at NMDA receptors [5]. Beyond its anesthetic use, ketamine shows promise in managing chronic pain, post-traumatic stress disorder (PTSD), and neurodegenerative diseases due to its anti-inflammatory properties and ability to modulate neural connectivity [6]. Ketamine also demonstrates rapid-acting antidepressant effects [7], and low-dose sublingual ketamine has been reported to be a safe and effective therapy for treatment-resistant depression (TRD) [8-12].

Ketamine has been studied for its role in interrupting central sensitization and reducing pain perception. By blocking NMDA receptors, ketamine inhibits the excitatory neurotransmission that contributes to central sensitization, thereby alleviating neuropathic pain [13]. Additionally, ketamine's sympatholytic effects can lead to vasodilation and improved microvascular perfusion. Studies have shown that ketamine induces direct vasodilation by reducing the intracellular calcium concentration in vascular smooth muscle cells, which promotes vasodilation [14]. These combined effects suggest ketamine may be a useful agent in the management of conditions associated with central sensitization and impaired microvascular perfusion. However, despite ketamine's potential therapeutic benefits, concerns about its abuse potential and adverse effects, such as dissociation and hemodynamic instability, have limited research on this medication [4,15].

Literature searches were conducted on PubMed, Google Scholar, Embase, and Cochrane Library using "ketamine treatment for Raynaud's phenomenon" as the search topic, and no published articles were found on any of these search engines. This article describes what is believed to be the first published case report in which improvement in primary Raynaud's phenomenon symptoms occurred in a patient being treated with low-dose sublingual ketamine.

## Case presentation

The patient is a 71-year-old female who both lives and receives treatment in Colorado. She presented to the clinic with a history of TRD, PTSD, attention-deficit hyperactivity disorder, and generalized anxiety disorder. Prior psychiatric treatment involved trials of numerous antidepressant medications, including citalopram, escitalopram, fluoxetine, sertraline, paroxetine, levomilnacipran, bupropion, venlafaxine, and desvenlafaxine. The patient failed to respond adequately to all of these treatments.

The patient's past medical history is significant for obstructive sleep apnea, hypertension, and moderately severe primary Raynaud's phenomenon. The patient's symptoms of Raynaud's included: (1) toes turned red and redness extended over the forefoot bilaterally with a quarter-sized white/blanched spot on the bottom of the left foot; (2) fingers and toes on both hands that felt colder than other body parts and colder than the hands and feet of others she was with; (3) dry skin on the ends of the toes; and (4) numbness of the toes and bottoms of the feet. She did not experience any seasonal variation in her Raynaud's symptoms. She had never been offered treatment for Raynaud's phenomenon.

Her family history is positive for depression and anxiety. Social history is significant for the patient, having attained a PhD and two master's degrees. She currently works as a psychotherapist. She does not use tobacco, cannabis, or recreational drugs and drinks alcohol only rarely.

Due to persistent depression and a failure to respond to multiple conventional antidepressants, the patient was started on ketamine HCl 25 mg daily at bedtime. The medicine was started in the month of February, which is winter in Colorado. The medicine was prescribed for the treatment of depression. Because neither the patient nor the physician were aware of ketamine's potential efficacy as a treatment for Raynaud's phenomenon, no pre-treatment symptom ratings or photos of hands or feet were obtained. The medicine was self-administered sublingually at home. The dose was subsequently titrated upward.

In late March of the following year, which was 13 months after starting treatment, the ketamine dose was increased to 125 mg daily. No adverse effects were experienced. In November of that same year, approximately 7.5 months after increasing the dose to 125 mg/day, the patient observed marked improvement in her symptoms of Raynaud's phenomenon. She first noted that her toes were not as red as they had been in the past. Subsequently, she observed additional changes in Raynaud's symptoms. Approximately 1.5 months after noting these changes, she rated her symptoms on a visual analog scale (VAS). The symptoms were rated for severity both prior to and after treatment with ketamine. Her scores indicated improvement in four of five symptoms of Raynaud's phenomenon. In order of magnitude from most to least improved, her ratings were as follows (% improvement shown in parentheses): reduction in skin color changes (77%), cold sensitivity (75%), skin texture changes (71%), numbness or tingling (63%), and pain or discomfort (0%) (Table 1).

**Table 1:** Raynaud's symptoms pre- and post-treatment with daily ketamine All scores are reported on a 0-10 visual analog scale; improvement is calculated using the formula: Improvement (%) = (Initial Score/Change) × 100

Symptom	Pre-treatment	Post-treatment	Improvement
1. Skin color changes	9	2	77%
2. Cold sensitivity	8	2	75%
3. Skin texture changes	7	2	71%
4. Numbness or tingling	8	3	71%
5. Pain or discomfort	0	0	0%

In addition to these changes in symptoms reported on the VAS, the patient also reported a change in the pattern of her symptoms. She explained that following treatment with ketamine, her Raynaud's symptoms were less frequent and less severe than they had been prior to beginning treatment with ketamine.

## Discussion

Current research on ketamine as a treatment for Raynaud's phenomenon is sparse, and its use is not widely standardized or approved for this condition. In addition, ketamine can have side effects at higher doses, including dissociation and sedation, and there is also a potential for misuse [16]. However, low-dose sublingual ketamine provides a relatively safe option for the therapeutic use of ketamine [8-12].

This patient showed significant improvement in four out of five symptoms of Raynaud's phenomenon following long-term treatment with low-dose sublingual ketamine. It is possible that this improvement was merely a result of the episodic nature of Raynaud's phenomenon, and her symptoms may have improved without treatment. However, in addition to symptomatic improvement, she also reported a change in her historical symptom pattern. She described her symptoms as occurring less frequently and being less severe after initiating treatment with ketamine. Based on ketamine's known mechanisms of action, which include vasodilation with improved microvascular perfusion [14], it is equally likely that this patient's symptoms improved as a result of ketamine's anti-inflammatory and vasodilatory effects.

The limitations of this case report include its subjective nature. No objective measurements were obtained to corroborate the subjective findings. Additionally, the ratings of symptom severity prior to initiating treatment with ketamine were performed retrospectively. Thus, results could be affected by recall bias [17]. The symptoms of Raynaud's phenomenon tend to be episodic rather than continuous [1]. It is possible that this patient's improvement would have occurred in the absence of treatment. However, her description of not only her symptoms but also her symptom pattern suggests this may not have been the case. The lack of control is a factor that limits the extrapolation of findings. Expectation bias [18] is not likely to have been a factor in the patient's improvement, as the patient was not aware of the possibility that ketamine might ameliorate her Raynaud's symptoms, nor was this discussed between the patient and her physician prior to initiating treatment with ketamine. Randomized, double-blind, placebo-controlled studies are needed to further investigate ketamine as a treatment for Raynaud's phenomenon. The safety, cost-effectiveness, and relevant mechanism of action of ketamine suggest that this medicine is an excellent candidate for further investigations.

## Conclusions

Low-dose sublingual ketamine provides a unique, safe option as a treatment for depression. Its vasodilatory properties suggest it might provide treatment for Raynaud's phenomenon. This case report describes a patient who experienced relief from the symptoms of Raynaud's phenomenon during treatment with low-dose sublingual ketamine. Furthermore, this patient's improvement occurred during the late fall months, suggesting that changes in ambient temperature were unlikely to have played a role in her improvement. While association does not prove causation, this case provides anecdotal evidence suggesting ketamine might have a role as a treatment for Raynaud's phenomenon. Randomized, double-blind, placebo-controlled studies are needed to investigate low-dose sublingual ketamine as a therapy for Raynaud's phenomenon. If these studies provide positive findings, additional studies designed to explore various doses and different lengths of treatment will be helpful in determining optimal treatment protocols.
